# Rheumatoid arthritis and risk of site-specific cancers: Mendelian randomization study in European and East Asian populations

**DOI:** 10.1186/s13075-022-02970-z

**Published:** 2022-12-13

**Authors:** Shuai Yuan, Jie Chen, Xixian Ruan, Mathew Vithayathil, Siddhartha Kar, Xue Li, Amy M. Mason, Stephen Burgess, Susanna C. Larsson

**Affiliations:** 1 Unit of Cardiovascular and Nutritional Epidemiology, Institute of Environmental Medicine, Karolinska Institutet, Nobelsväg 13, 17 177 Stockholm, Sweden; 2 Centre for Global Health, Zhejiang University School of Medicine, 866 Yuhangtang Road, Hangzhou, China; 3 Department of Gastroenterology, The Third Xiangya Hospital, Central South University, 138 Tongzipo Road, Changsha, China; 4 MRC Cancer Unit, University of Cambridge, Cambridge, UK; 5 MRC Integrative Epidemiology Unit, Bristol Medical School, University of Bristol, Bristol, UK; 6 Department of Big Data in Health Science School of Public Health, Center of Clinical Big Data and Analytics of The Second Affiliated Hospital, Zhejiang University School of Medicine, Hangzhou, China; 7 British Heart Foundation Cardiovascular Epidemiology Unit, Department of Public Health and Primary Care, University of Cambridge, Cambridge, UK; 8 MRC Biostatistics Unit, University of Cambridge, Cambridge, UK; 9 Department of Public Health and Primary Care, University of Cambridge, Cambridge, UK; 10 Unit of Medical Epidemiology, Department of Surgical Sciences, Uppsala University, Uppsala, Sweden

**Keywords:** East Asian, European, Mendelian randomization, Rheumatoid arthritis

## Abstract

**Background:**

The associations of rheumatoid arthritis (RA) with risk of site-specific cancers beyond lymphohematopoietic cancer have been scarcely explored. We conducted a Mendelian randomization investigation of the associations of RA with site-specific cancers in European and East Asian populations.

**Methods:**

Independent genetic variants strongly associated with RA in European and East Asian populations were selected as instrumental variables from genome-wide association studies of 58,284 European individuals (14,361 cases and 43,923 controls) and 22,515 East Asian individuals (4873 cases and 17,642 controls), respectively. The associations of genetic variants with overall and 22 site-specific cancers were extracted from the UK Biobank study (*n* = 367,561), the FinnGen study (*n* = 260,405), Biobank Japan (*n* = 212,453), and international consortia. The associations for one outcome from different data sources were combined by meta-analysis.

**Results:**

In the European population, the combined odds ratios per 1-unit increase in log odds of genetic liability to RA were 1.06 (95% confidence interval [CI] 1.03–1.10) for head and neck cancer, 1.06 (95% CI 1.02–1.10) for cervical cancer, 0.92 (95% CI 0.87–0.96) for testicular cancer, and 0.94 (95% CI 0.90–0.98) for multiple myeloma. In the East Asian population, the corresponding odds ratios were 1.17 (95% CI 1.06–1.29) for pancreatic cancer, 0.91 (95% CI 0.88–0.94) for breast cancer, and 0.90 (95% CI 0.84–0.96) for ovarian cancer. There were suggestive associations for breast and ovarian cancer and overall cancer in the European population. No other associations were observed.

**Conclusion:**

This study suggests that RA may play a role in the development of several site-specific cancers.

**Supplementary Information:**

The online version contains supplementary material available at 10.1186/s13075-022-02970-z.

## Background

Rheumatoid arthritis (RA) is a prevalent chronic autoimmune disease with a high disease burden globally [1]. RA patients have severe comorbidities, including major mental disorders, lung disease, cardiovascular disease, and solid malignancies [2]. Epidemiological studies found that RA was associated with increased risk of overall cancer [3] and several site-specific cancers, such as lymphoma [3, 4], lung cancer [5], leukemia [6, 7], and cervical cancer [8], but with decreased risk of breast and colorectal cancer [3]. A recent Mendelian randomization (MR) study found that high levels of tumor necrosis factor, a clinical feature of RA patients, were associated with decreased risk of breast and colorectal cancers [9]. However, the inverse associations of RA with breast and colorectal cancer were not observed in other studies [10, 11]. The associations of RA with other site-specific cancers have been scarcely investigated. In addition, the majority of previous evidence on the associations between RA and cancer risk was based on observational studies where results are prone to be influenced by confounding and reverse causality. Thus, the causal role of RA in the development of cancer remains unestablished.

Mendelian randomization (MR) is an epidemiological approach that utilizes genetic variants as an instrument to strengthen causal inference [12]. MR is by nature not prone to confounding since genetic variants are randomly assorted at conception and thus unrelated to environmental and self-adopted factors. Additionally, this method can minimize reverse causality since germline genotypes are unaffected by the onset and progression of the disease. A recent MR study found a protective association of genetic liability to RA and breast cancer risk in an East Asian population [13]. However, the MR associations of RA with risk of site-specific cancers have not been explored in a systematic way. Here, we conducted an MR investigation to determine the associations of RA with risk of overall cancer and site-specific cancers in European and East Asian populations.

## Materials and methods

### Study design

**Fig. 1:**
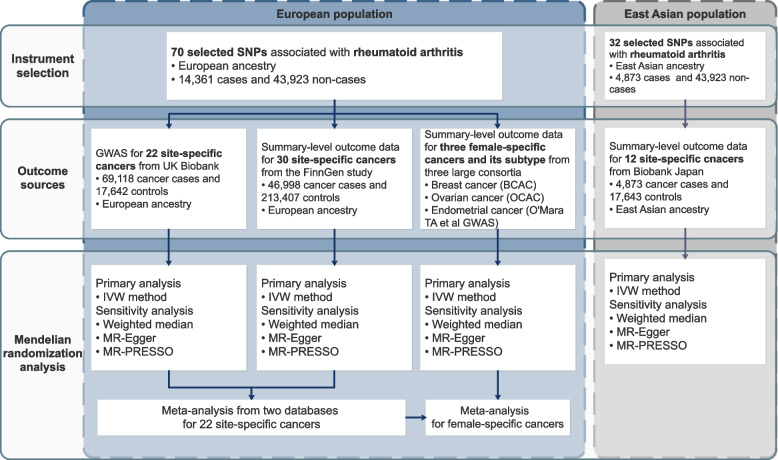
Overview of the present study design. BCAC, Breast Cancer Association Consortium; IVW, inverse variance weighted; MR-PRESSO, Mendelian randomization pleiotropy residual sum and outlier; OCAC, the Ovarian Cancer Association Consortium; SNP, single-nucleotide polymorphism

An overview of the study design is presented in Fig. 1. The present study is based on data on RA and cancers from the UK biobank [14], the FinnGen study [15], the Biobank Japan [16], and genome-wide association studies (GWASs) on female-specific cancers [17–19] (Supplementary Table 1). All studies had been approved by a relevant ethical review board and participants had given informed consent. The present MR analyses were approved by the Swedish Ethical Review Authority (2019-02793).

### Cancer data sources

Genetic associations with cancers in European ancestry were obtained from the UK Biobank [14], FinnGen study [15], and international consortia, including the Breast Cancer Association Consortium (BCAC, 122,977 cases and 105,974 controls) [17], the Ovarian Cancer Association Consortium (OCAC, 25,509 cases and 40,941 controls) [18], and a genome-wide meta-analysis on endometrial cancer (12,906 cases and 108,979 controls, including UK Biobank) [19]. The definition of site-specific cancers in UK Biobank and FinnGen is displayed in Supplementary Table 2. Detailed descriptions on UK Biobank, FinnGen, and Biobank Japan [16], like exclusion criteria and quality control information, are presented in Supplementary Method.

### Genetic instrument selection

Single-nucleotide polymorphisms (SNPs) associated with RA in the European and East Asian populations at the genome-wide significance level (*P* < 5 × 10^−8^) were separately derived from a GWAS with a total of 58,284 European individuals (14,361 RA cases and 43,923 controls) and 22,515 East Asian individuals (4873 RA cases and 17,642 controls) [20]. After removal of SNPs with linkage disequilibrium (*r*^*2*^ ≥ 0.01), 70 and 32 SNPs were selected as instrumental variables in the analysis in the European and East Asian populations, respectively (Supplementary Table 3). Odds ratios (ORs) and corresponding confidence intervals (CIs) of the associations were scaled to a 1-unit increase in log-transformed odds of genetic liability to RA in the main analysis.

To provide estimates with a more intuitive interpretation, we estimated absolute genetic associations with RA using linear regression with adjustment for age, sex, and first 10 genomic principal components among the UK Biobank participants [21]. RA was defined using electronic health records (ICD-9 714.0, ICD-10: M05 or M06). These summary-level coefficients were used in the sensitivity analysis where the OR of cancer was scaled to per 1% increase in the probability of RA. Based on these estimates obtained from linear regression, the phenotypical variance explained by used RA-associated SNPs was estimated to be approximately 0.3%, which was used in *F* statistic and power calculation for the analysis in the European population. Estimates of SNPs in the sensitivity analysis are given in the Supplementary Table 3.

### Statistical analysis

All instrumental variables for each outcome were harmonized to omit ambiguous SNPs with non-concordant alleles and palindromic SNPs with ambiguous minor allele frequency (> 0.42 and < 0.58). If an SNP was unavailable in the outcome data, a proxy SNP in high linkage disequilibrium (*r*^*2*^ ≥ 0.8) will replace. We found no evidence of pleiotropic associations of RA-associated SNPs with cancer by a search in the PhenoScanner platform [22].

The primary analysis was conducted based on the multiplicative random-effects inverse-variance weighted method [23]. The weighted median [23], MR-egger regression [24], and Mendelian randomization pleiotropy residual sum and outlier (MR-PRESSO) [25] methods were performed as sensitivity analyses. The weighted median model can provide valid estimates if at least 50% of the weight comes from valid instrumental variables [23]. MR-Egger method can provide estimates after adjusting for pleiotropy; however, the obtained associations are usually imprecise [24]. MR-PRESSO method can detect outliers of SNPs with pleiotropic effects and provide estimates after removal of outliers [25]. Heterogeneity among estimates of SNPs was assessed by the Cochran’s *Q* value. The intercept test of MR-Egger regression was used to identify horizontal pleiotropy [24]. The fixed-effects meta-analysis was used to combine MR estimates from different data sources for each site-specific cancer. Power of the analysis in the European population was estimated using an online tool (Supplementary Table 4) [26]. The Benjamini-Hochberg method that controls the false discovery rate (FDR) was applied to correct for multiple testing. The association with a Benjamini–Hochberg adjusted *P*-value < 0.05 was deemed significant. The association with a nominal *P*-value < 0.05 and a Benjamini–Hochberg adjusted *P*-value ≥ 0.05 was treated as a suggestive association. All analyses were two-sided and performed using the TwoSampleMR [27] and MRPRESSO [25] packages in R software 4.1.2.

## Results

### Genetic instruments

Seventy and thirty-two RA-associated SNPs were available in the UK Biobank study and Biobank Japan, respectively. One SNP was unavailable and without a suitable proxy SNP, leaving 69 SNPs as an instrumental variable in the FinnGen study. All *F* statistics for the analyses in the European populations were above 10 (Supplementary Table 4).

### RA and cancer in the European population

**Fig. 2:**
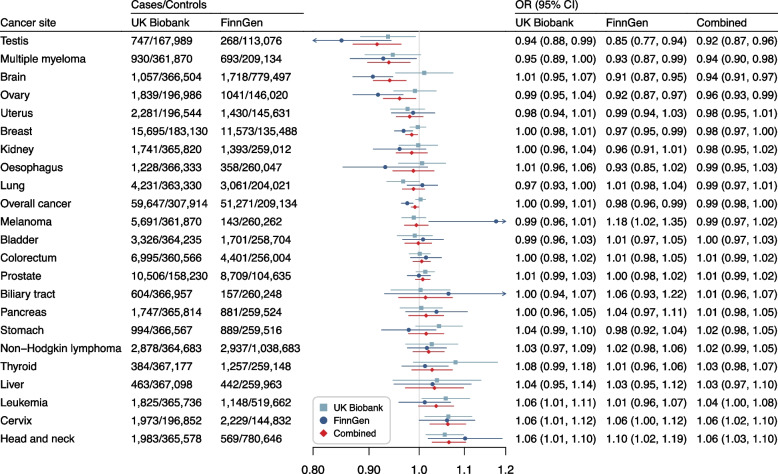
Associations of genetic liability to rheumatoid arthritis with risk of overall cancer and 22 site-specific cancers in European individuals in the UK Biobank and FinnGen studies. CI, confidence interval; OR, odds ratio

The associations of genetic liability to RA with overall cancer and 22 site-specific cancers are displayed in Fig. 2. Genetic predisposition to RA was not associated with 22 studied site-specific cancers or overall cancer in the UK Biobank study after Benjamini–Hochberg correction (Fig. 2, Supplementary Table 5, and Supplementary Table 6). The direction of the associations in UK biobank was overall consistent with that in the FinnGen study (Fig. 2). However, genetic predisposition to RA showed significant associations with a decreased risk of cancers of brain, testis, breast, and ovary as well as overall cancer in the FinnGen study (Fig. 2, Supplementary Table 6, and Supplementary Table 7). We detected high heterogeneity but no horizontal pleiotropy in the analysis of overall cancer. The association for overall cancer remained in the MR-PRESSO analysis after removal of the outliers (OR 0.98, 95% CI 0.97-0.99) (Supplementary Table 7).

In the combined analysis of UK Biobank and FinnGen, genetic liability to RA was nominally associated with an increased risk of head and neck cancer (OR 1.06, 95% CI 1.03–1.10) and cervical cancer (OR 1.06, 95% CI 1.02–1.10), and a decreased risk of testicular cancer (OR 0.92, 95% CI 0.87–0.96), multiple myeloma (OR 0.94, 95% CI 0.90–0.98), brain cancer (OR 0.94, 95% CI 0.91–0.97), ovarian cancer (OR 0.96, 95% CI 0.93–0.99), breast cancer (OR 0.98, 95% CI 0.97–1.00), and overall cancer (OR 0.99, 95% CI 0.98–1.00) (Fig. 2). The associations for cancers of brain, head and neck, testis, and uterus, and multiple myeloma remained after Benjamini–Hochberg adjustment for multiple comparisons (Supplementary Table 6). In the sensitivity MR analysis using instrumental variables with summary-level coefficients from linear regression, the association between genetic liability to RA and overall cancer and 22 site-specific cancers showed the same pattern with a larger magnitude of the estimates (Supplementary Table 8). The association between genetic liability to RA and the risk of leukemia became clear. Per 1% increase in genetic liability to RA, the combined OR of leukemia was 1.07 (95% CI 1.01–1.14).

**Fig. 3:**
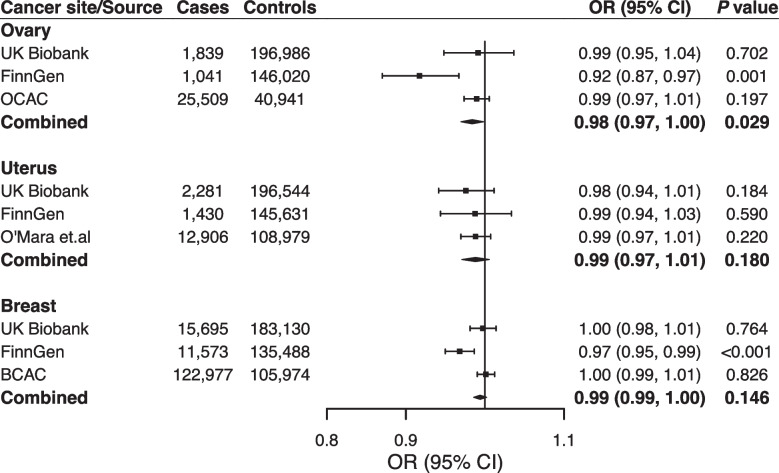
Associations of genetic liability to rheumatoid arthritis with risk of female-specific cancers in European individuals in the UK Biobank, FinnGen, and international consortia. BCAC Breast Cancer Association Consortium; CI, confidence interval; OCAC, the Ovarian Cancer Association Consortium; OR, odds ratio

We did not observe any associations for female-specific cancers in the analyses based on consortia data (Supplementary Table 9). In the meta-analysis combining data from UK Biobank, FinnGen, and consortia, there was a suggestive association between genetic liability to RA and ovarian cancer (OR 0.98, 95% CI 0.97–1.00) (Fig. 3). No associations were observed with cancer of the breast or uterus in this combined analysis (Fig. 3).

### RA and cancer in the East Asian population

**Fig. 4:**
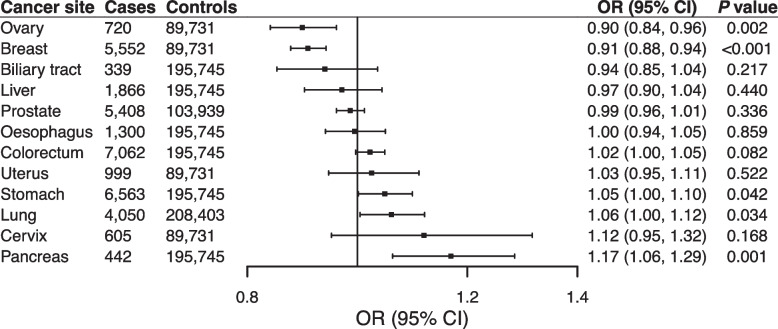
Associations of genetic liability to rheumatoid arthritis with risk of 12 site-specific cancers in East Asian individuals in the Biobank Japan. CI, confidence interval; OR, odds ratio

The OR per 1-unit increase in genetically predicted log odds of RA was 1.17 (95% CI 1.06–1.29) for pancreatic cancer, 0.91 (95% CI 0.88–0.94) for breast cancer, and 0.90 (95% CI 0.84–0.96) for ovarian cancer (Fig. 4). The results remained consistent in the sensitivity analyses (Supplementary Table 10). We observed heterogeneity in the analysis of breast cancer and the association became stronger after removal of one outlier SNP. We detected possible horizontal pleiotropy in MR-Egger intercept analysis of pancreatic cancer and no outliers in the corresponding MR-PRESSO analysis (Supplementary Table 10). There were no associations of genetic liability to RA with the other studied cancers (Fig. 4).

## Discussion

In the current MR study, we found that genetic liability to RA was associated with an increased risk of cancer of head and neck and cervix and possibly leukemia and a decreased risk of brain and testicular cancer, multiple myeloma, and possibly breast and ovarian cancer in the European population. Genetic liability to RA was associated with an increased risk of pancreatic cancer and a decreased risk of breast and ovarian cancer in the East Asian population.

The identified associations between RA and female-specific cancers are in line with previous studies. A cohort study comprising 58,979 American women with RA found that the risk of high-grade cervical dysplasia and cervical cancer was 1.5 times higher in individuals with RA [8]. In a nationwide register-based cohort study including 374,944 Swedish females, biologic-naïve women with RA were at greater risk of cervical intraepithelial neoplasia (CIN) 1 (hazard ratio 1.53, 95% CI 1.23–1.89) and CIN 2–3 (hazard ratio 1.39, 95% CI 1.16 to 1.66) [28]. A possible explanation for this positive association is that RA patients’ autoimmunity disorders may lead to a deficient response to human papillomavirus infection, which ultimately results in invasive cervical cancer. Regarding breast cancer, previous observational studies have consistently found a lower risk of breast cancer in RA patients than in women without this disease [29–31]. An MR study based on data from the BCAC, Biobank Japan, and Consortium of Investigators of Modifiers of BRCA1/2 also found that genetically proxied RA liability based on 25 SNPs was inversely associated with breast cancer in the East Asian population [13]. Nevertheless, this inverse association was not observed in the European population in our analysis. The reason that may explain this ethnicity-specific association remains unknown. Our study further demonstrated RA to be associated with a decreased risk of ovarian cancer. More studies are needed to verify this association.

The biological mechanism behind the observed associations between genetic liability to RA and decreased risk of breast and ovarian cancers is unclear. Female hormonal factors may play an essential role. Studies on serum sex hormone levels in RA patients found that pre-menopausal RA women had lower concentrations of luteinizing hormone [32] and high levels of luteinizing hormone in pre-menopausal women may result in the immature release of the ovum, which may contribute to improper re-epithelialization of the ovaries [33]. MicroRNA-498 was found in lower levels in peripheral blood of RA patients [34], and it is suspected to be associated with the development of breast cancer [35]. Likewise, polymorphisms in the *DRB1* gene, the primary genetic susceptibility locus for RA [36], have recently been linked to a decreased risk of breast cancer [37]. The estradiol levels were similar between female RA patients’ and controls [32, 38], and endometrial cancer is strongly related to estradiol levels, which may explain our null finding for endometrial cancer.

The present MR results did not support observational studies suggesting an elevated risk of overall and lung cancer and reduced risk of colorectal cancer among RA patients. A meta-analysis of 23 studies found that the standardized incidence ratio in RA patients compared with the general population was 1.09 (95% CI 1.06–1.13) for overall cancer, 1.64 (95% CI 1.51–1.79) for lung cancer, and 0.78 (95% CI 0.71–0.86) for colorectal cancer [3]. A cohort study found that the standardized incidence ratio (RA patients vs. controls) for colorectal cancer was 0.78 (95% CI 0.68–0.91) [39]. In a retrospective cohort study including 1885 Korean RA patients, the risk of overall cancer and lung cancer was higher in all RA patients and male RA patients, respectively [10]. A recent meta-analysis of 11 cohort studies involving 183,888 patients also found that having RA was associated with a higher lung cancer risk [5]. However, our findings are consistent with an MR study where there was a null association of genetic liability to RA with risk of lung cancer and its subtypes in a European population [5]. The discrepancies between our results and previous observational findings might be caused by residual confounding (e.g., smoking habits, drug use) or reverse causation bias in the observational studies.

In this MR study, we observed non-significant positive associations of genetic liability to RA with non-Hodgkin lymphoma and leukemia risk, thereby partly supporting most observational studies which have found an increased risk of lymphohematopoietic cancer in RA patients [3, 4, 6, 7, 10, 11, 40]. A possible explanation for this positive association is that most patients with RA are treated with methotrexate, biologics, or disease-modifying antirheumatic drugs, all of which might contribute to the development of lymphohematopoietic cancer. The non-significant associations in the present MR analysis might be explained by inadequate power caused by a small number of cases. In contrast to observational findings, we observed an inverse association between RA and multiple myeloma. In a meta-analysis comprising 18 observational studies, a slightly increased (14% higher) risk of multiple myeloma was observed in RA patients [41]. However, significant heterogeneity and confounding factors remained as the primary limitations of this study, which suggests caution in interpretation of the results.

We found that genetic liability to RA was positively associated with head and neck cancer risk. Epidemiological data on this association are limited. A retrospective cohort study in the USA showed that patients treated with tumor necrosis factor inhibitor did not have a significantly increased risk of head and neck cancer recurrence [42]. However, female RA patients were found to have a higher risk of buccal cavity/pharynx cancer compared to healthy controls [43]. Evidence for the pathogenic effect of chronic immune stimulation/chronic inflammation suggests that RA itself could lead to the increased risk of tumor formation [44]. Furthermore, RA results in the number and function of T-suppressor lymphocytes decreasing, which may impair the ability to direct against the pro-oncogenic virus including Epstein-Barr virus and papillomavirus [45]. We also observed inverse associations for testicular and brain cancers. These are novel findings that need confirmation.

We found a positive association between genetic liability to RA and pancreatic cancer risk in East Asian individuals. Previous observational findings on this association were inconsistent. In a nationwide Japanese cohort, the incidence of pancreatic cancer in female patients with RA was slightly lower than in the general population, but the potential confounders were not adjusted for [39]. A null association between RA and pancreatic cancer was observed in another study of the Asian population [4]. Thus, the role of RA in the development of pancreatic cancer in East Asians needs more investigation.

A chief strength of this study is MR design with data from different populations, which diminishes confounding and reverse causality as well as examines the ancestral difference in the associations. We performed MR analysis in the European and East Asian populations, separately, which minimized bias caused by population structure bias.

A major limitation of the current MR study was that several site-specific cancers had relatively small numbers of cases, which resulted in low precision in MR estimation and possible false negative findings, given that used SNPs for RA explained a small phenotypic variance. Another disadvantage is that we could not completely rule out horizontal pleiotropic effects that might influence our findings even though we conducted a series of sensitivity analyses that indicated limited pleiotropic effects. A further limitation is that the MR analysis assessed the life-long liability to RA. Thus, our findings might not be compared to the effect of RA on cancer during a particular lifecycle. There was a large sample overlap between the exposure and outcomes in the analysis in the East Asian population, which might cause weak instrument bias [46]. Given lack of information on variance explained by used instruments in the East Asian population, *F*-statistic was not able to be estimated to measure this bias. Finally, our results might not be comparable with those of observational studies as RA status was defined by genetic liability based on genetic variants in MR analysis.

In conclusion, this study suggests that RA may play a role in the development of several site-specific cancers. Future studies are warranted to verify our findings.

## Supplementary Information

**Additional file 1: Supplementary Method.** Detailed description on UK Biobank, FinnGen, and Biobank Japan. **Supplementary Table 1.** Information of included studies and consortia. **Supplementary Table 2.** Definition site-specific cancers in UK Biobank and FinnGen. **Supplementary Table 3.** SNPs used as instrumental variable for rheumatoid arthritis in European ancestry and East Asian populations. **Supplementary Table 4.** Power calculations for overall and 22 site-specific cancers. **Supplementary Table 5.** Associations of genetic predisposition to rheumatoid arthritis with site-specific cancers in the primary inverse-variance weighted analysis and in sensitivity analyses using other Mendelian randomisation methods in UK Biobank. **Supplementary Table 6.** False discovery rate adjusted *p* values for all tested associations. **Supplementary Table 7.** Associations of genetic predisposition to rheumatoid arthritis with site-specific cancers in the primary inverse-variance weighted analysis and in sensitivity analyses using other Mendelian randomisation methods in FinnGen study. **Supplementary Table 8.** Associations of genetic predisposition to rheumatoid arthritis with site-specific cancers In the sensitivity MR analysis using instrumental variables with summary-level coefficients from linear regression. **Supplementary Table 9.** Associations of genetic predisposition to rheumatoid arthritis with women-related site-specific cancers in the primary inverse-variance weighted analysis and in sensitivity analyses using other Mendelian randomisation methods in large consortium. **Supplementary Table 10.** Associations of genetic predisposition to rheumatoid arthritis with site-specific cancers in the primary inverse-variance weighted analysis and in sensitivity analyses using other Mendelian randomisation methods in Biobank Japan.

## Data Availability

Data used can be obtained from a reasonable request to the corresponding author.

## Abbreviations

BCAC: The Breast Cancer Association Consortium
CI: Confidence interval
CIN: Cervical intraepithelial neoplasia
FDR: False discovery rate
GWAS: Genome-wide association studies
ICD: International Classification of Diseases
MR: Mendelian randomization
MR-PRESSO: Mendelian randomization pleiotropy residual sum and outlier
OCAC: The Ovarian Cancer Association Consortium
OR: Odds ratio
RA: Rheumatoid arthritis
SNP: Single-nucleotide polymorphism
