# Supplementary material for: Rheumatoid arthritis and risk of site-specific cancers: Mendelian randomization study in European and East Asian populations
Source: Arthritis Res Ther. 2022 Dec 13;24:270. doi: 10.1186/s13075-022-02970-z (PMC9746148; doi:10.1186/s13075-022-02970-z)

Supplementary information for

**Rheumatoid arthritis and risk of site-specific cancers: Mendelian randomization study in European and East Asian populations**

*Shuai Yuan, Jie Chen, Xixian Ruan, Mathew Vithayathil, Siddhartha Kar, Xue Li, Amy M. Mason, Stephen Burgess, Susanna C. Larsson*

| **Supplementary method**. Detailed descritpion on UK Biobank, FinnGen, and Biobank Japan |
| --- |
| **Supplementary Table 1**. Information of included studies and consortia |
| **Supplementary Table 2**. Definition site-specific cancers in UK Biobank and FinnGen |
| **Supplementary Table 3**. SNPs used as instrumental variable for rheumatoid arthritis in European ancestry and East Asian populations |
| **Supplementary Table 4**. Power calculations for overall and 22 site-specific cancers |
| **Supplementary Table 5**. Associations of genetic predisposition to rheumatoid arthritis with site-specific cancers in the primary inverse-variance weighted analysis and in sensitivity analyses using other Mendelian randomisation methods in UK Biobank |
| **Supplementary Table 6**. False discovery rate adjusted p values for all tested associations |
| **Supplementary Table 7**. Associations of genetic predisposition to rheumatoid arthritis with site-specific cancers in the primary inverse-variance weighted analysis and in sensitivity analyses using other Mendelian randomisation methods in FinnGen study |
| **Supplementary Table 8**. Associations of genetic predisposition to rheumatoid arthritis with site-specific cancers In the sensitivity MR analysis using instrumental variables with summary-level coeffients from linear regression |
| **Supplementary Table 9**. Associations of genetic predisposition to rheumatoid arthritis with women-related site-specific cancers in the primary inverse-variance weighted analysis and in sensitivity analyses using other Mendelian randomisation methods in large consortium |
| **Supplementary Table 10**. Associations of genetic predisposition to rheumatoid arthritis with site-specific cancers in the primary inverse-variance weighted analysis and in sensitivity analyses using other Mendelian randomisation methods in Biobank Japan |

Above supplementary tables can be found in <https://osf.io/c3qdj/>


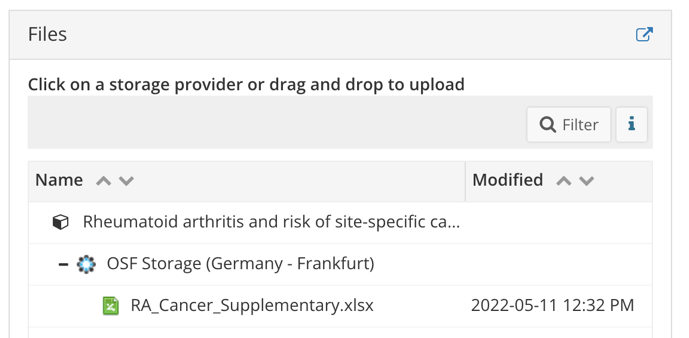

Supplement: Supplementary file 1 — Additional file 1: Supplementary Method. Detailed description on UK Biobank, FinnGen, and Biobank Japan. Supplementary Table 1. Information of included studies and consortia. Supplementary Table 2. Definition site-specific cancers in UK Biobank and FinnGen. Supplementary Table 3. SNPs used as instrumental variable for rheumatoid arthritis in European ancestry and East Asian populations. Supplementary Table 4. Power calculations for overall and 22 site-specific cancers. Supplementary Table 5. Associations of genetic predisposition to rheumatoid arthritis with site-specific cancers in the primary inverse-variance weighted analysis and in sensitivity analyses using other Mendelian randomisation methods in UK Biobank. Supplementary Table 6. False discovery rate adjusted p values for all tested associations. Supplementary Table 7. Associations of genetic predisposition to rheumatoid arthritis with site-specific cancers in the primary inverse-variance weighted analysis and in sensitivity analyses using other Mendelian randomisation methods in FinnGen study. Supplementary Table 8. Associations of genetic predisposition to rheumatoid arthritis with site-specific cancers In the sensitivity MR analysis using instrumental variables with summary-level coefficients from linear regression. Supplementary Table 9. Associations of genetic predisposition to rheumatoid arthritis with women-related site-specific cancers in the primary inverse-variance weighted analysis and in sensitivity analyses using other Mendelian randomisation methods in large consortium. Supplementary Table 10. Associations of genetic predisposition to rheumatoid arthritis with site-specific cancers in the primary inverse-variance weighted analysis and in sensitivity analyses using other Mendelian randomisation methods in Biobank Japan. [file 13075_2022_2970_MOESM1_ESM.docx]
